# COVID-19, jobs and skills—Exploratory analysis of the job postings in the US and UK healthcare job market

**DOI:** 10.1371/journal.pone.0278237

**Published:** 2023-01-20

**Authors:** Himanshu Upadhyay, Mecit Can Emre Simsekler, Maher Maalouf, Siddiq Anwar, Mohammed Omar

**Affiliations:** 1 Department of Industrial and Systems Engineering, Khalifa University of Science and Technology, Abu Dhabi, United Arab Emirates; 2 Department of Medicine, Sheikh Shakbout Medical City, Abu Dhabi, United Arab Emirates; University of Georgia, UNITED STATES

## Abstract

The COVID-19 pandemic has significantly affected all spheres of life, including the healthcare workforce. While the COVID-19 pandemic has started driving organizational and societal shifts, it is vital for healthcare organizations and decision-makers to analyze patterns in the changing workforce. In this study, we aim to identify patterns in healthcare job postings during the pandemic to understand which jobs and associated skills are trending after the advent of COVID-19. Content analysis of job postings was conducted using data-driven approaches over two-time intervals in the pandemic. The proposed framework utilizes Latent Dirichlet Allocation (LDA) for topic modeling to evaluate the patterns in job postings in the US and the UK. The most demanded jobs, skills and tasks for the US job postings are presented based on job posting data from popular job posting websites. This is obtained by mapping the job postings to the jobs, skills and tasks defined in the O*NET database for the healthcare occupations in the US. The topic modeling results clearly show increased hiring for telehealth services in both the US and UK. This study also presents an increase in demand for specific occupations and skills in the USA healthcare industry. The results and methods used in the study can help monitor rapid changes in the job market due to pandemics and guide decision-makers to make organizational shifts in a timely manner.

## 1. Inroduction

The advent of COVID-19 was completely unexpected and it has overwhelmed and strained the healthcare system in many countries [1]. This led to a paradigm shift in how healthcare was delivered to cater to the unexpected increase in demand. According to the Future of Work report by the World Economic Forum, two trends in healthcare during the pandemic are the increase in demand for digital transformation specialists and training and development specialists [1]. This can be attributed to the digitization of healthcare services during the pandemic [2]. The job vacancies reduced significantly during the second half of March, 2020 in the US. By late April, 2020 job postings fell by 40% in the US. The US job posting data showed a significant decline in job postings in all industries, including healthcare [3]. During the COVID-19 pandemic, various organizations pivoted to telemedicine and had no choice but to create the supporting infrastructure. Current and evolving telecommunication technologies can help overcome physical barriers to facilitate the secure exchange of medical information to diagnose and manage diseases.

The primary modalities for remote consultations include telephone consultations, virtual fracture clinics and video consultations [4–6]. These innovations could be a disruptive influence on how health care will be delivered in the future. This would further increase the demand for people who can help maintain such secure platforms. Hence there can be an increase in job postings mentioning this type of task in the healthcare sector. The COVID-19 pandemic has led to a surge in the use of telemedicine for urgent care and non-urgent care visits beyond baseline periods [7, 8]. The COVID-19 pandemic has been a disruptive influence on health care provisions. Even legacy healthcare systems have been forced to re-evaluate their operational practices. Research has been conducted on analyzing job postings using data science tools in the past [9, 10]. However, these studies have analyzed data from one time period in the past; therefore, there is a need to analyze the healthcare job market conditions during the pandemic in different time periods. This study contributes towards previous studies by analyzing data in two time periods during the pandemic and comparing the patterns towards hiring over time and geographies. Using topic modeling, a content analysis study can provide good insights into the job posting data [11, 12]. Hence, we not only present topic modelling, but also present ways to find most in demand jobs and skills during a certain period of time. This research presents a comprehensive study guided by the following questions:

1. What are the trending keywords in the job postings and what do these keywords tell about the types of trending jobs?
2. What is the difference in the content of job postings in the US and UK healthcare industry?
3. What are the most trending O*NET healthcare occupations, skills, and tasks during the pandemic based on the US job posting data?

## 2. Materials and methods

### 2.1 Data collection

The data used in this study were collected from various job posting websites. After an exploratory analysis to find the best websites that contain job postings, we collected data from several web pages such as Indeed.com and Monster.com. For data collection, a web scraping technique was used with the help of libraries available in Python. Scraping is a useful tool to collect data from websites. To ensure data diversification, multiple job posting websites for data collection were considered such that the collected data is a good representation of the overall job market demand. We collected data from the US and the UK job posting websites.

The data was collected over two different time intervals. Period 1 of data collection includes a timeframe from 1 July 2020 to 15 September 2020, and period 2 of data collection includes a timeframe from 1 October 2020 to 1 December 2020. Data were collected regularly to check for saturation and duplications in each period. The number of US job postings collected in period 1 was 2642 and in period 2 was 2500. The number of UK job postings collected in period 1 was 3771. Data from different periods of COVID-19 were collected to observe any changes in the demand pattern for jobs in the healthcare industry in different periods of the pandemic. Data from two different countries were collected to compare the observed patterns.

We collected O*NET jobs data from the August 2020 release. The data collected was then cleaned to include only healthcare jobs. A total of 190 occupation descriptions are there in the healthcare and social assistance category of the O*NET database. Table 1 presents the summary of the data characteristics.

**Table 1:** Data collection.

Period	Country	Period	# of job posting
1	US	1 July—15 September, 2020	2642
1	UK	1 July—15 September, 2020	3771
2	US	1 October—1 December, 2020	2500

### 2.2 Data pre-processing

The data collection in each period in Table 1 was separately conducted to go through pre-processing by following the standard steps as described below:

1. Tokenization: We started the pre-processing with tokenization of the job postings data. Tokenization is used to split the text in the collected data into smaller entities called tokens—where tokens can be anything from individual words to paragraphs or sentences.
2. Removing English Stop-Words: English Stop-Words (e.g., and, or, to, as, a, the) are almost present in every text corpus and do not provide any additional information. In addition, these words unnecessarily increase the matrix size, hence they were deleted in the pre-processing step.
3. Additional Filtering: Overfitting is a common problem when the clustering or classification function fits too firmly on training data. To overcome this issue, additional filtering was conducted; therefore, words that appear in more than 90% of the documents (occupations) and words that are too rare and appear only in 10% of the documents were deleted.
4. Lemmatization: The last step was to remove morphological affixes from words, leaving only the base dictionary form of the words, a process commonly known as Lemmatization.
5. TF-IDF: This refers to the Term Frequency-inverse document frequency. This is used to filter out words that are not relevant to the document. This method was used to create a dictionary of only relevant words. This method can also shorten the number of words used in defining topics.

The document contained a considerable amount of words, which we filtered to 4000 terms only.

### 2.3 Latent dirichlet allocation and topic modeling

Latent Dirichlet Allocation is a generative probabilistic model for collecting discrete data such as text corpora [13]. This work was further improved to develop the relational topic model (RTM) [14], which besides LDA, unveils the links between document contents. In this study, LDA is solely required because it uses Dirichlet distribution [15] which supposes that all occupations cover only a small set of topics.

The topics are described by a minimal set of words. Implementing LDA is usually done with two approaches: 1) Using an Expectation Maximization (EM)-like procedure called variational inference [16]; and 2) Using a randomized algorithm called Gibbs sampling [17]. To summarize, LDA helps discover which topics are present in any document by observing all the words and generating a topic distribution. Hence, Latent Dirichlet Allocation (LDA) was deployed for topic modeling in healthcare job posting data.

For a given set of parameters α and β, the joint distribution of a topic mixture θ, a set of N topics z, and a set of N words w are given by:

$$p\left(\theta ,z,w|\alpha ,\beta \right)=p\left(\theta |\alpha \right)\overset{N}{\underset{n=1}{\prod }}p\left(z_{n}|\theta \right)p\left(w_{n}|z_{n},\beta \right)$$ (1)

where *p*(*z*_*n*_|*θ*) is equal to θ_i_ for the unique i such that $z_{n}^{i}=1$.

Integrating over θ and summing over z, the marginal distribution of a document is obtained:

$$p\left(w|\alpha ,\beta \right)=\int p\left(\theta |\alpha \right)\left(\overset{N}{\underset{n=1}{\prod }}\underset{z_{n}}{\sum }p\left(z_{n}|\theta \right)p\left(w_{n}|z_{n},\beta \right)\right)d\theta$$ (2)

Finally, using marginal probabilities of single documents, the probability of a corpus is obtained. The value of α can be adjusted. The higher the value of α, the higher the number of topics in the document. The value of α is set low to keep the number of topics small. In the same way, the value of parameter β can be adjusted. The higher value of β signifies more number of words representing each topic. We keep this value lower as otherwise, it will become too complicated to get the distinction between topics. So we set both the values on the lower side to get better results.

Another important step is the selection of the number of topics. For this, we used the Coherence score. This score is used to determine the topics’ interpretability since LDA learns topics in an unsupervised way. The higher the coherence score for the model, the topics should be more intuitive. Coherence was calculated from the following formula:

$$C\left(t;V^{\left(t\right)}\right)=\;\overset{M}{\underset{m=2}{\sum }}\overset{m-2}{\underset{l=1}{\sum }}\text{log}\frac{D\left(v_{m}^{\left(t\right)},v_{l}^{\left(t\right)}\right)+1}{D\left(v_{l}^{\left(t\right)}\right)}$$ (3)

where *D*(*v*) denotes the document frequency of word type *v* and *D*(*v*, *w*) denotes the co-document frequency of word types *v* and *w*. Also, $V^{\left(t\right)}=\;\left(v_{1}^{\left(t\right)},v_{2}^{\left(t\right)},\ldots ..v_{M}^{\left(t\right)}\right)$ is the list of M’s most probable words in topic t.

**Fig 1:**
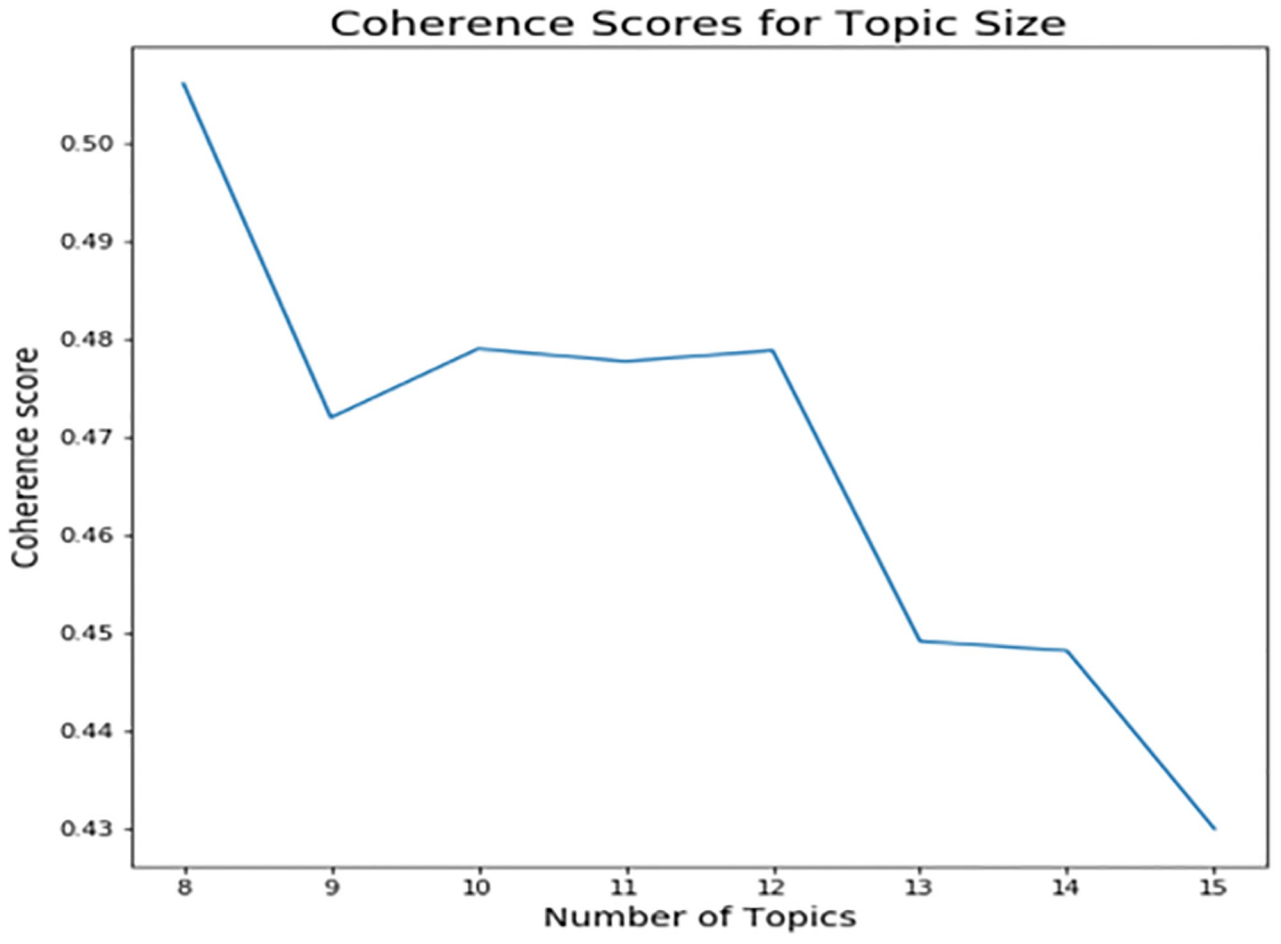
Coherence score for US job posting data.

**Fig 2:**
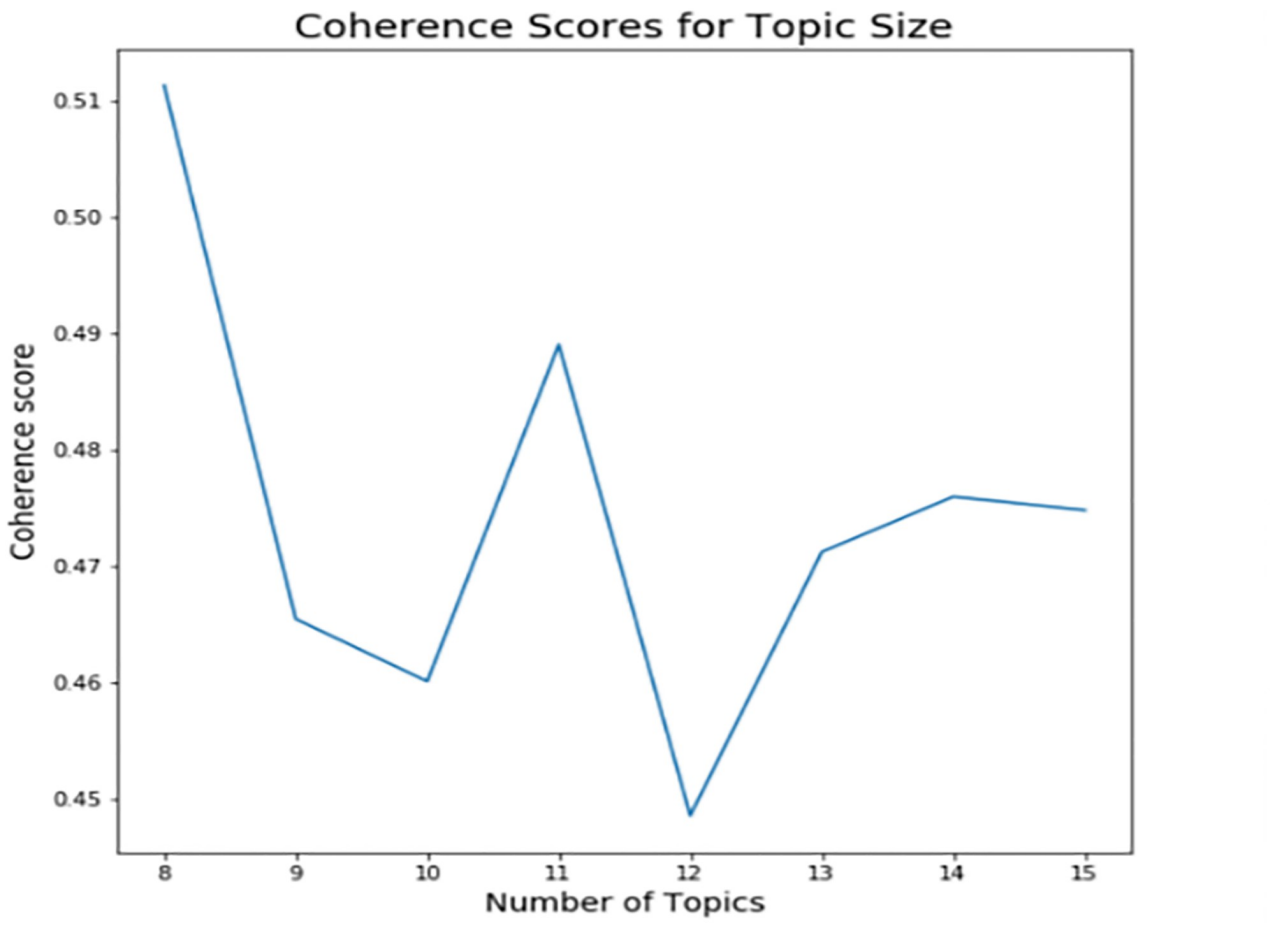
Coherence score for UK job posting data.

The coherence score vs. the number of topics was plotted. The plots are presented for the US and the UK data of Period 1 separately in Figs 1 and 2, respectively.

The highest coherence for both US and UK data was achieved at eight topics, as shown in Figs 1 and 2, respectively. The coherence score dropped below 0.5 for less than eight topics, and it continued dropping for a higher number of topics. Finally, eight topics were the optimal number of topics. Also, the number of topics below eight can be inconvenient for identifying a broad range of topics in healthcare data, so we did not consider coherence scores below eight.

**Fig 3:**
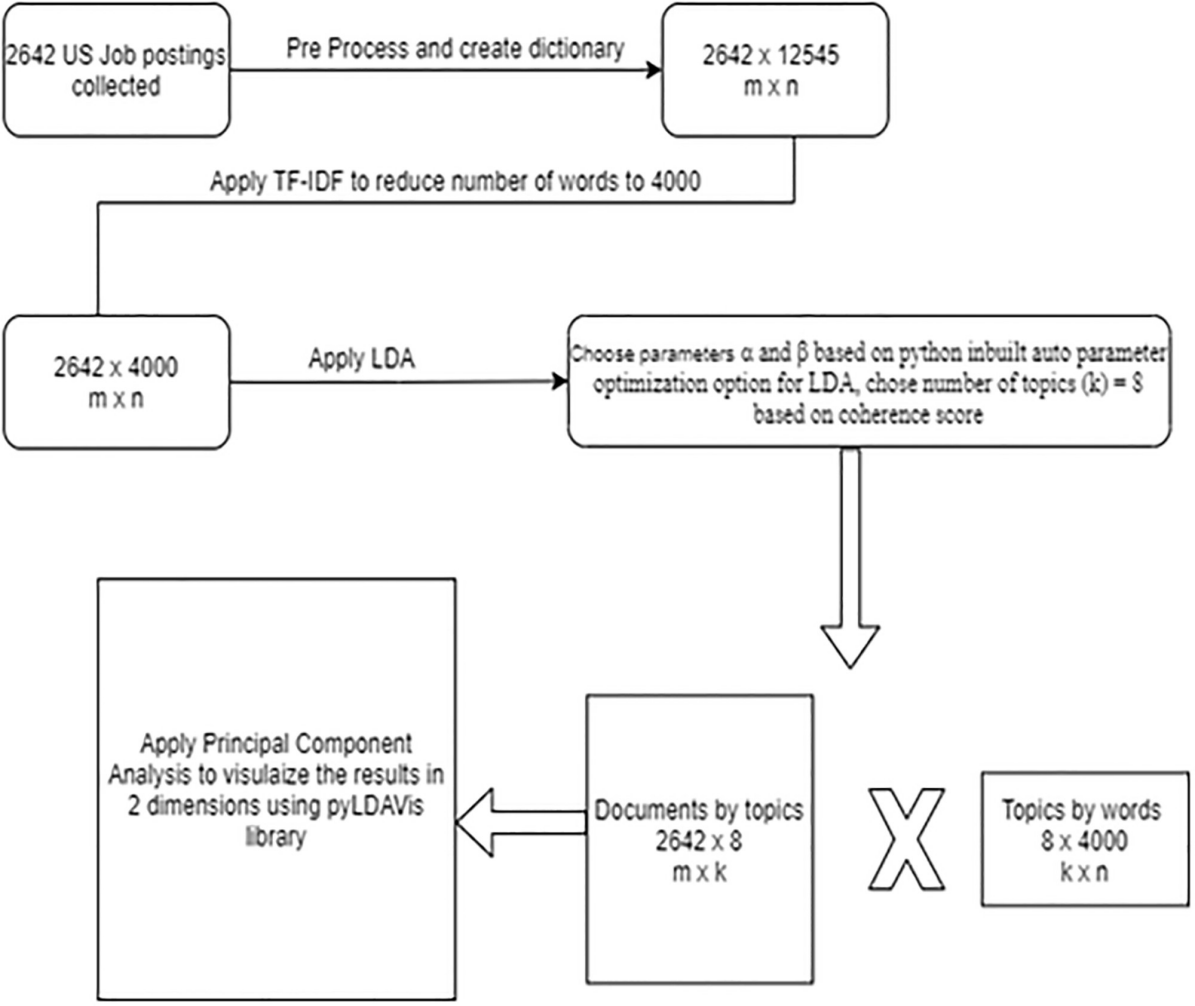
Schematic representation of the methodology used for topic modeling.

Visualization of topics was done using the pyLDAvis library. pyLDAvis utilizes Principle Component Analysis (PCA) to reduce the number of dimensions and help users easily interpret the topics in a topic model that fits a corpus of text data. The schematic diagram of the methodology used for topic modeling for US data in period 1 is provided in Fig 3. For all the topic modeling same methodology was used, and results after PCA are presented and discussed in the next section.

### 2.4 Mapping job postings to O*NET occupations, skills and tasks separately

This step maps the collected job postings to the O*NET database. This will ensure that the job postings are standardized according to the O*NET format. Hellinger distance [18] was applied to calculate the similarity weights. Since the output of the LDA model gives probability distributions, Hellinger distance is a common choice because it is used for measuring the difference between two probability distributions. Given two discrete probability distributions, P and Q, Hellinger distance is defined as:

$$H\left(P,Q\right)=\frac{1}{\sqrt{2}}\times \sqrt{\sum_{i=1}^{k}{\left(\sqrt{p_{i}}-\sqrt{q_{i}}\right)}^{2}}$$ (4)

The Hellinger distance is a value between 0 and 1, and the higher the value, the less the similarity. To make interpretation better, we subtract the values from 1 so that the larger Hellinger distance values signify larger similarities, which will lead to negative weights. The final correction is applied as follows (see Eq 5):

$$\hat{H}\left(P,Q\right)=1-H\left(P,Q\right)$$ (5)

**Fig 4:**
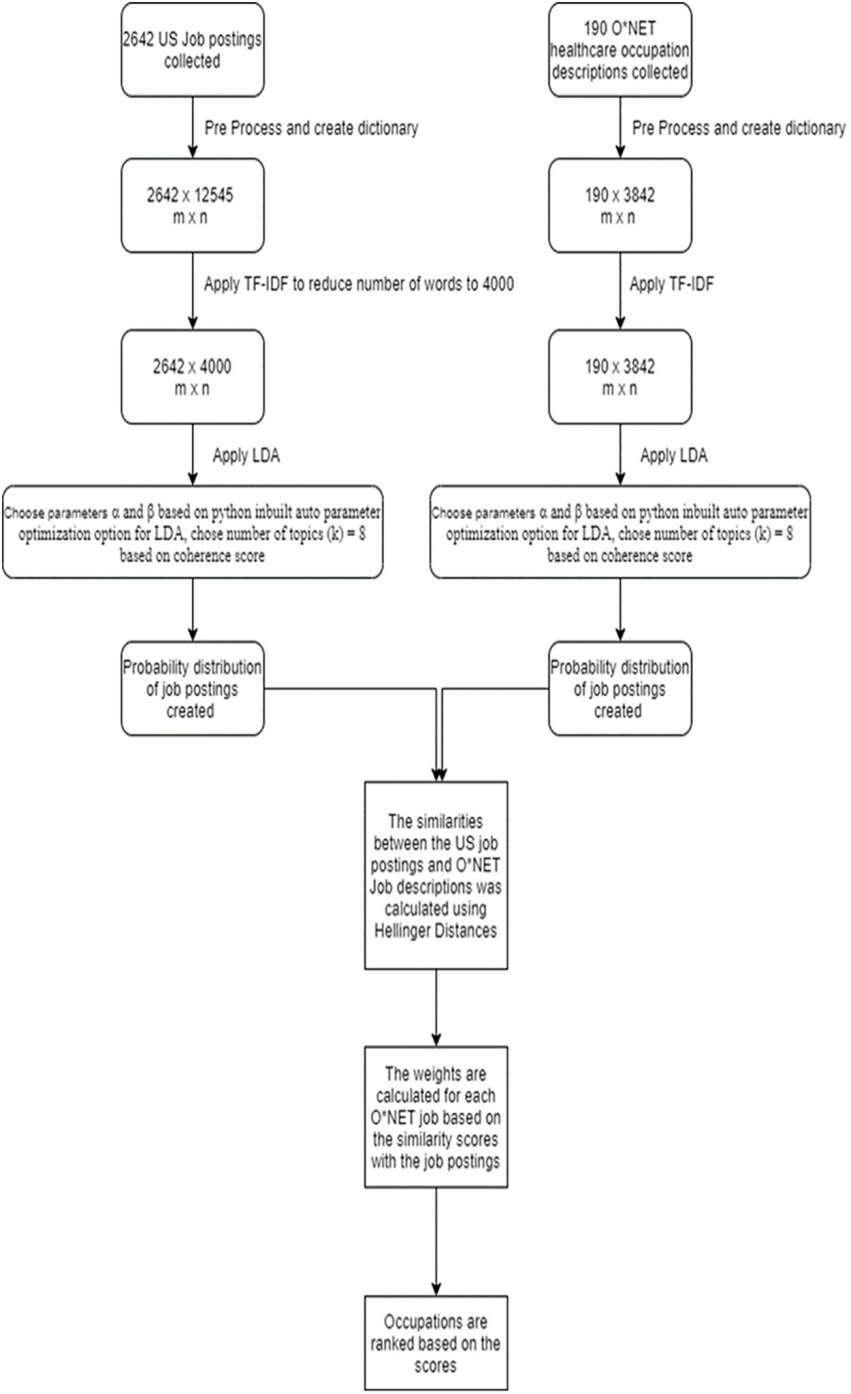
Schematic representation of the methodology used for Approach 1.

Each US job posting was projected into the corpus of documents created by the O*NET database fields. Three approaches were used in this study:

1. The corpus is produced by the occupation descriptions of the 190 healthcare occupations in the O*NET database. See Fig 4.
2. The corpus is produced by combining the fields, skills elements name, and skills work activities name of the O*NET database. Every step is similar to the one in the previous approach and the weights were calculated for each 232 skills elements name and skills work activities combined.
3. The corpus is produced by the detailed work activities field of healthcare occupations. Every step is similar to the first approach and the weights were calculated for each 827 detailed work activities in the O*NET database.

## 3. Results

### 3.1 Topic modeling results for raw US and UK job postings without standardization

The topic modeling for US and UK data was done to get a preliminary analysis of the collected job postings. The job postings were collected during the pandemic; hence the topic modeling can present preliminary hiring patterns in the US and UK healthcare job market.

The salient features in a topic model can be using to interpret a possible topic in a corpus of documents. Previous studies have used topic modeling to identify topics of discussion in healthcare on social media. Author and expert judgement is used to interpret words in the salient terms [19]. We will use similar techniques to interpret results of the topic model.

**Fig 5:**
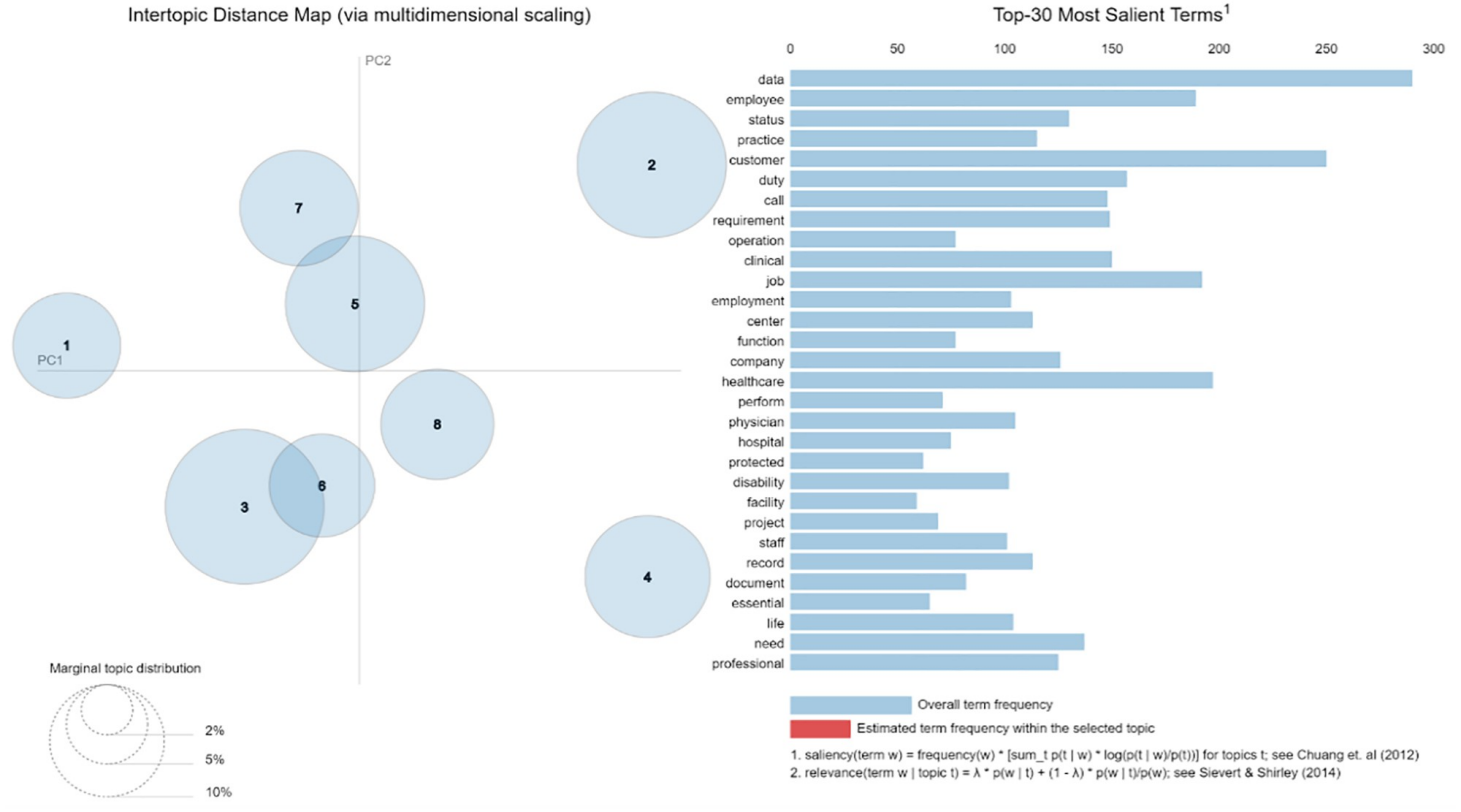
US topic modeling results (Period 1).

As shown in Fig 5, the overall corpus of documents created from US job postings in period 1 has data as one of the most salient features. Furthermore, words like call and center point to an increase in telehealth services required by various hospitals in the US. The rest of the words seem more generic, and these topics represent different fields in the healthcare industry.

The top three most significant topics are selected to determine which part of the healthcare industry jobs they represent using our domain knowledge. The larger the circle, the more relevant the topic is in the job postings’ overall corpus. This can indicate the type of jobs in demand during the pandemic in the US healthcare sector.

The top three topics for period 1, with the possible fields representing them, are presented in Table 2. Topic 3 reinforces the fact that there has been an increase in telehealth services during the pandemic, and hence more jobs mention these keywords in job postings in the US.

**Table 2:** US data top three topics (Period 1).

Topic No.	Most salient terms	Potential representative field
2	Status, employment, provider, protected, equal, disability, veteran, employer	HR department terms
3	Call, duty, appointment, record, office, scheduling, customer, schedule	Telehealth services, call center services
5	Data, business, project, management, analysis, report, technology	Data Analytics and Management

Further, topic 5 shows an incline toward collecting and managing data during the pandemic. Again, these results reinforce the studies that suggest an increase in data analytics and AI in the healthcare industry during the pandemic. The top three topics for period 2, with the possible fields representing them, are presented in Table 3.

**Table 3:** US data top three topics (Period 2).

Topic No.	Most salient terms	Potential representative field
2	health, status, care, employment, opportunity, working, gender, equal, disability, veteran	HR department terms
7	Provider, customer, Call, duty, appointment, record, office, scheduling, phone	Telehealth services, call center services
3	duty, job, record, information, office, computer, responsibility, maintain, physical, review	Not any specific division

According to Table 3, telehealth was still in high demand during Period 2, but the data analytics jobs decreased. This can be attributed to a decrease in social distancing and tracking measures initially started by the US government as a prompt response to COVID-19. However, data analytics was still a significant part of job postings in the second data collection period. Telehealth seems to have impacted healthcare in the long term [20].

**Fig 6:**
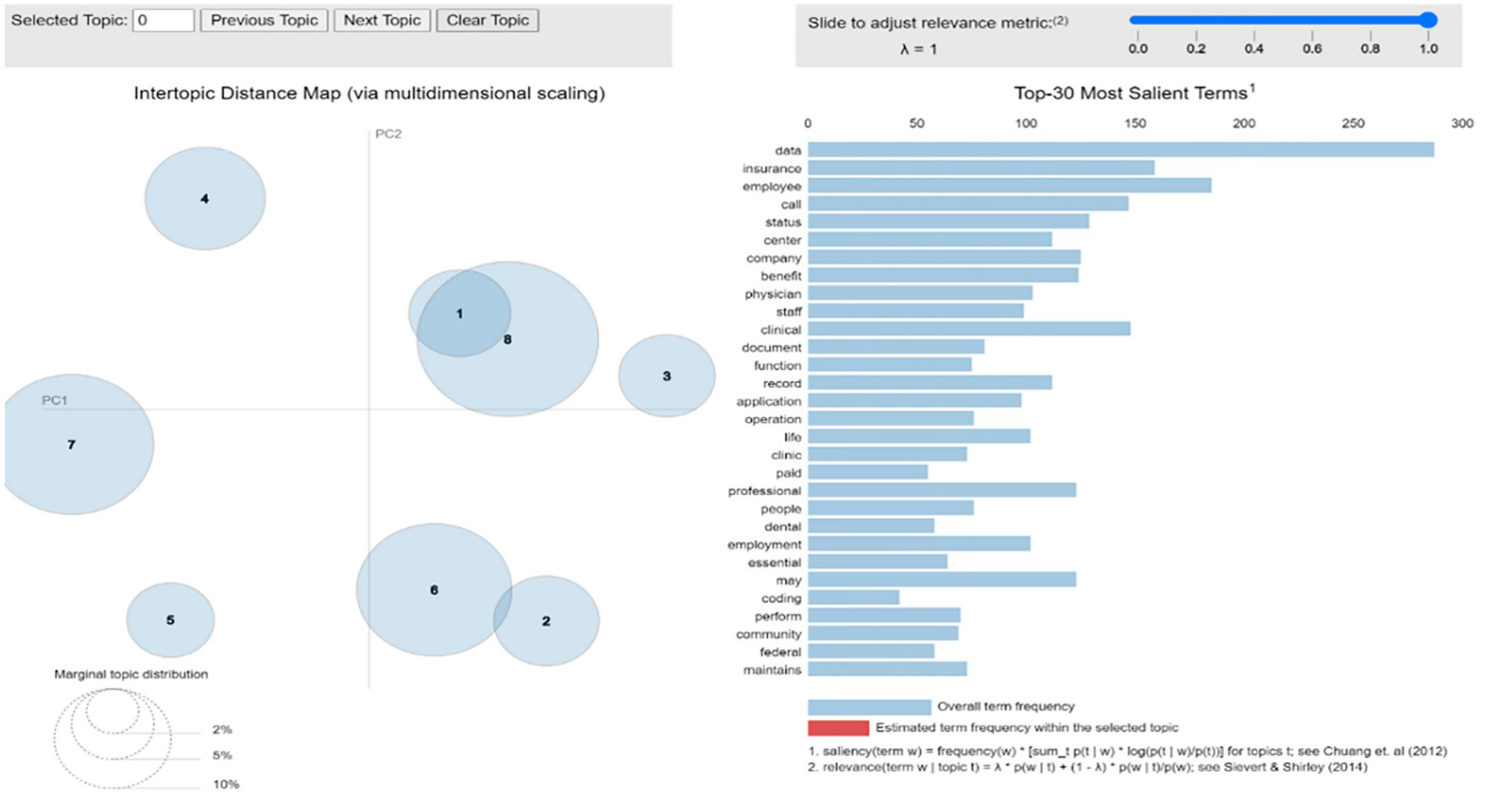
UK topic modeling results (Period 1).

The covid-19 pandemic has brought a revolution in the integration of telehealth in general healthcare services. This justifies the increase in job postings related to telehealth services in our study’s periods 1 and 2. The topic modeling results for the UK healthcare job postings data are presented in Fig 6.

The most salient terms from the UK job postings were related to either analytics or telehealth services like data and call. Insurance also is a quite relevant term as it may point to possible fear in people for their health [3].

The insurance data collected by the study in the US points to a similar hiring pattern. However, in our data, it was not reflected while conducting topic modeling for the US data, and it may be because the data collected is not as extensive as is required. The top three topics for period 1 of the UK data with the possible fields representing them are presented in Table 4.

**Table 4:** UK data top three topics (Period 1).

Topic No.	Most salient terms	Potential representative field
6	Data, business, project, improvement, analyst, analysis, tool, report, technology	Data Analytics
7	Status, employment, member, provider, protected, gender, equal	HR department terms
8	Call, schedule, appointment, phone, insurance, representative	Telehealth, consulting, insurance services

According to Table 4, topics 6, 7 and 8 provide similar results to the US data in period 1 of the pandemic. Hence both the regions went through a similar situation regarding job vacancies in the healthcare sector during period 1 of data collection. The period 2 data for the UK was not used for this study.

### 3.2 Results for US job postings mapped to O*NET database occupations (Approach 1)

After initial analysis, the second type of analysis involved standardizing the US job postings using the O*NET database. The standardization was done using a similarity function called Hellinger distances. With the help of weights generated, we identified the top in-demand jobs, skills and tasks in the US healthcare job market. The O*NET database combines healthcare and social assistance into one industry. However, we eliminated the social assistance occupations for our study.

The top 15 occupations obtained based on weights obtained from periods 1 and 2 data are provided in Table 5.

**Table 5:** Top 15 in-demand occupations in the US healthcare sector.

Top 15 in-demand occupations in Period 1	Top 15 in-demand occupations in Period 2
1. Therapists, All Other	1. Healthcare Support Workers, All Other
2. Physicians and Surgeons, All Other	2. Personal Care and Service Workers, All Other
3. Communications Equipment Operators, All Other	3. Health Technologists and Technicians, All Other
4. Healthcare Support Workers, All Other	4. Health Diagnosing and Treating Practitioners, All Other
5. Respiratory Therapy Technicians	5. Training and Development Managers
6. Psychiatric Technicians	6. Medical and Health Services Managers
7. Licensed Practical and Licensed Vocational Nurses	7. Audiologists
8. Respiratory Therapists	8. Human Resources Specialists
9. Anesthesiologist Assistants	9. Radiologists
10. Medical and Health Services Managers	10. Dermatologists
11. Radiologic Technologists	11. Physical Therapists
12. Dietetic Technicians	12. Pathologists
13. Medical Secretaries	13. Cytogenetic Technologists
14. Psychiatric Aides	14. Community Health Workers
15. Medical Records and Health Information Technicians	15. Dietitians and Nutritionists

The top occupations show a remarkable feature: increased demand for respiratory therapy technicians and therapists, which can be due to COVID-19 causing respiratory issues. Further, in the top 15, we can see Medical Records and Health Information technicians, which reinforces our findings without standardizationand shows the high frequency for data collection and analysis words. Previous studies on most in-demand jobs using 2017 to 2018 data showed different trends in the occupation’s demands [10], thus it is clear there were drastic changes during the pandemic. The sudden increase in demand for respiratory therapists and respiratory therapy technicians was the primary feature. Another significant change, compared to previous studies [10], is the high demand for communication equipment operators, which justifies the high rise in telehealth services during the pandemic [2].

The increase in demand for healthcare support workers can be attributed to the continuous increase in COVID-19 cases in the US. To manage the patients, an increase in support staff is required. There is also an increase in the hiring of service managers as more practitioners got hired, so more managers were required to manage them. The high rise in demand for health services managers and training and development managers is an entirely new observation compared to previous studies [10].

#### 3.2.1 Results for US job postings mapped to O*NET database skills and work activities combined (Approach 2)

We got weights for various skills and work activities combined in this case. The top 10 skills and work activities for periods 1 and 2 are presented in Table 6 in decreasing order of their weights:

**Table 6:** Top 10 skills in Periods 1 and 2.

Top 10 skills in Period 1	Top 10 skills in Period 2
1. Technology Design, Thinking Creatively:	1. Writing Documenting/Recording Information
2. Coordination Assisting and Caring for Others	2. Monitoring Provide Consultation and Advice to Others
3. Coordination Developing and Building Teams	3. Coordination Scheduling Work and Activities
4. Negotiation Developing and Building Teams	4. Persuasion Provide Consultation and Advice to Others
5. Service Orientation Assisting and Caring for Others	5. Speaking Provide Consultation and Advice to Others
6. Programming Interacting with Computers	6. Coordination Provide Consultation and Advice to Others
7. Monitoring Assisting and Caring for Others	7. Service Orientation Assisting and Caring for Others
8. Speaking Developing and Building Teams	8. Speaking Developing and Building Teams
9. Monitoring Developing and Building Teams	9. Speaking Communicating with Supervisors, Peers, or Subordinates
10. Writing Documenting/Recording Information	10. Mathematics Processing Information

The skills in demand point out to more service orientation and soft skills required by the employers. Points 6 and 10 shows a pattern towards data collection using computers and a demand for programming skills even in the healthcare sector. The collection and recording of data seem to be the most demanded skillset in period 2 of data collection. The increase in demand for consultation skills can be attributed to an increase in healthcare consultancy services during the later period of the Covid-19 pandemic.

#### 3.2.2 Results for US job postings mapped to O*NET database detailed work activities (Approach 3)

The top 5 detailed work activities for the US healthcare sector in period 1 are presented in Table 7.

**Table 7:** Top 5 in-demand DWAs in the US healthcare sector.

Period 1 DWAs	Period 2 DWAs
1. Assist others during emergencies.	1. Verify the accuracy of records
2. Prepare accident or incident reports.	2. Confer with clients to exchange information
3. Supervise scientific or technical personnel	3. Instruct workers to use equipment or perform technical procedures
4. Code data or other information.	4. Collect biological specimens
5. Prepare biological samples for testing or analysis	5. Assist other educational professionals with projects or research

The results from Detailed work activities did not seem very exciting, but they mainly represented an emergency and collection of data. The topic modeling results for the top 2 quartiles of data represented a pattern in hiring more for data analytics and telehealth, reinforcing the earlier findings. The top 5 detailed work activities for the US healthcare sector in period 2 are also presented in Table 7.

In data analysis from period 2, we found an increase in the collection of biological specimens as a task. This signifies the increase in testing rates in the later period of the pandemic. Also, the data collected during the pandemic needs to be verified. So many people who got hired in this period were expected to perform data verification.

The data used in this study and the source code is published on the following github link to facilitate the reproducibility of the results: Please also see Supplement for all data sources used in this study.

https://github.com/me2140733/Healthcare-Jobs-Analysis-Post-COVID-19

## 4. Discussions

This study aims to understand the impact of COVID-19 on the US and UK healthcare job market. We aimed to study the skills and jobs in high demand during the first phase of the pandemic. We looked to study the US and UK job market conditions during the pandemic and see the differences. There is extensive literature on various techniques to determine the skills and occupations in demand during a specific period. However, a framework through which the job market condition can be monitored during pandemics and crises was still unavailable. This study contributes in addressing this gap.

We gathered more than 2000 job postings over two periods in the COVID-19 pandemic from various job posting websites. We designed a step-wise analysis framework to provide insights into rapid changes in the healthcare job market during the COVD-19 pandemic. Below, the most important findings are discussed further:

The raw job posting data analysis indicated a remarkable increase in hiring for telehealth during the pandemic in both the US and UK job markets. This is further confirmed by surveys on how hospitals use telehealth during the pandemic [4–6].

This study confirms the radical shift which occurred during the pandemic to pivot the outpatient services to telehealth. The telehealth job postings dominated periods 1 (1 July 2020 to 15 September 2020) and period 2 (1 October to 1 December) of our study. Adopting telehealth in a significant way has the potential to transform chronic disease management through telehealth and remote monitoring technologies [2, 20]. The COVID-19 pandemic not only resulted in a drastic increase in telehealth services for both urgent and non-urgent care but also increased remote working and video meetings [7, 8].

This study also provided some remarkable results after standardization. The increase in demand for respiratory therapists in period 1 suggests the effect of COVID-19 on the US healthcare job market. Also, there is an increase in the need for soft skills and man-management due to the rapid increase in the workforce to cope with the pandemic and ensure operational readiness. In period 2, we noticed an increase in support workers’ job postings due to the number of cases and the increased burden of managing the number of patients in the USA. Several health-related occupations, such as Nurses, Medical Equipment Preparers and Healthcare Social Workers were employed [21].

Our study indicated that in phase 1, there was a demand for skills sets such as data collection, programming and computer-related skills. As the pandemic was a great unknown, most healthcare organizations wanted to make better healthcare decisions by studying the considerable amount of new data generated. In period 2, the increase in demand for consultation skills was quite evident. In response to the COVID-19 pandemic, health care modalities such as video consultations have been rapidly developed to provide safe health care and minimize the risk of spread [22].

Our analysis also suggests that the healthcare sector sees increased demand for data analytics and information processing. Better data gathering and creating structured data sets will allow the implementation of AI-driven algorithms for precise predictions and readings in the future [23].

Despite the increase in healthcare demand during the pandemic, the total number of job postings has reduced overall [3], shifting towards requiring analytics skills. This suggests that healthcare institutions were looking to optimize their processes to utilize existing healthcare resources to cater to increased healthcare demand. Hence there is an excellent need for existing healthcare workers to upgrade their skills to meet the requirements of their organization. During the COVID-19 pandemic, agencies and organizations have leveraged the power of ’information’ and utilized their ’business knowledge’ for sustainability and future-proofing the organization to cope with the economic downturn [23]. The job postings in healthcare may also be due to workers falling ill during the pandemic, but as we only analyzed the increase in demand of certain types of jobs and skills within the healthcare domain, this aspect will not have any significant effect on our analysis.

The limitations of this study are the following. This study has a few limitations as the data collected does not cover the whole pandemic extensively. This study touches on just five months period (1 July 2020 to 1 December 2020) of the pandemic, and hence future work can be done based on the overall pandemic data. Also, comparative analysis, using the presented framework before and after the pandemic, can provide further insights into the disruption in healthcare delivery triggered by Covid-19. This study does not make any pre-assumptions and aims to visualize the patterns in job postings during the pandemic. This study does not consider the jobs which are already filled up. Hence further studies using surveys targeted at employers can be deployed to get an overall picture of the job market.

## 5. Conclusion

In the current unpredictable times of the pandemic, studying the occupational data patterns becomes very important to future-proof organizations. The data provided by O*NET is undoubtedly an essential source of information for organizations to track changes in the job market over time, but this does not account for very rapid changes like the ones we are witnessing nowadays. Hence there is a need to study the current hiring pattern in the job market using real-time data. This study proposed a Latent Dirichlet Allocation (LDA) model that utilizes O*NET occupational descriptions, tasks, skills, and raw job postings to identify the most demanded occupations in the healthcare job market for the US and UK.

This study provides more refined results than US official sources like O*NET, as the changes are not made very often to these databases. In addition, the proposed methodology can be applied to any geography and any industry. Although this is just a preliminary study for visualizing hiring pattern in the job market, this can be further improved using surveys and expert opinions. Various validation methods can be incorporated to supplement the findings using data analytics techniques. In future work, we aim to compare the pre-pandemic and post-pandemic data to get better insights into the impact of COVID-19 on the healthcare industry. It should be noted that new APIs have recently been developed, that future studies can benefit from using that data to create a real-time pipeline that will help decision-makers and policymakers make decisions in a timely manner.

## Supporting information

S1 File (XLSX)
